# The Archaeal Small Heat Shock Protein Hsp17.6 Protects Proteins from Oxidative Inactivation

**DOI:** 10.3390/ijms22052591

**Published:** 2021-03-04

**Authors:** Pengfei Ma, Jie Li, Lei Qi, Xiuzhu Dong

**Affiliations:** 1 State Key Laboratory of Microbial Resources, Institute of Microbiology, Chinese Academy of Sciences, Beijing 100101, China; mapengfei006@gmail.com (P.M.); lijie824@im.ac.cn (J.L.); qil@im.ac.cn (L.Q.); 2 College of Life Sciences, University of Chinese Academy of Sciences, No.19A Yuquan Road, Shijingshan District, Beijing 100049, China

**Keywords:** small heat shock protein, Archaea, antioxidation, aspartate oxidation

## Abstract

Small heat shock proteins (sHsps) are widely distributed among various types of organisms and function in preventing the irreversible aggregation of thermal denaturing proteins. Here, we report that Hsp17.6 from *Methanolobus psychrophilus* exhibited protection of proteins from oxidation inactivation. The overexpression of Hsp17.6 in *Escherichia coli* markedly increased the stationary phase cell density and survivability in HClO and H_2_O_2_. Treatments with 0.2 mM HClO or 10 mM H_2_O_2_ reduced malate dehydrogenase (MDH) activity to 57% and 77%, whereas the addition of Hsp17.6 recovered the activity to 70–90% and 86–100%, respectively. A similar effect for superoxide dismutase oxidation was determined for Hsp17.6. Non-reducing sodium dodecyl sulfate polyacrylamide gel electrophoresis assays determined that the Hsp17.6 addition decreased H_2_O_2_-caused disulfide-linking protein contents and HClO-induced degradation of MDH; meanwhile, Hsp17.6 protein appeared to be oxidized with increased molecular weights. Mass spectrometry identified oxygen atoms introduced into the larger Hsp17.6 molecules, mainly at the aspartate and methionine residues. Substitution of some aspartate residues reduced Hsp17.6 in alleviating H_2_O_2_- and HClO-caused MDH inactivation and in enhancing the *E. coli* survivability in H_2_O_2_ and HClO, suggesting that the archaeal Hsp17.6 oxidation protection might depend on an “oxidant sink” effect, i.e., to consume the oxidants in environments via aspartate oxidation.

## 1. Introduction

Small heat shock proteins (sHsps) are a family of stress-inducible protein chaperones. They possess a conserved α-crystallin domain and have molecular weights ranging from 12 to 43 kDa and usually form oligomers comprising 9 to 50 subunits [1,2,3,4,5]. sHsps are ubiquitously distributed in all types of organisms, including bacteria, archaea, and eukaryotes, and play significant roles in protecting proteins from thermal aggregation and in mitigating the irreversible aggregation of denaturing proteins. 

sHsps are typically complexed with a variety of denaturing proteins in an ATP-independent manner, and act as the first line of defense against protein aggregation in response to elevated temperatures, oxidation, bacterial infection-caused inflammation, etc., and maintain protein homeostasis. Therefore, sHsps are considered as important components in the protein quality control network.

Research has determined that the human sHsps, such as Hsp20, showed multifunctional effects in cardioprotection, anti-apoptosis, inhibition amyloid formation, and possibly in carcinogenesis, cell division, and differentiation [6,7,8,9,10,11]. Bacterial sHsps also function in assisting the hosts to cope with adverse environments. Kandror and Goldberg [12] found that induced expression of the *Escherichia coli* sHsps was harmful and reduced the cell viability when growing at 4 °C. Ferrer et al. [13] showed that overexpression of the Cpn60/10 gene from *Oleispira antarctica,* a psychrophilic bacterium from the Antarctic, increased *E. coli* growth rates by 3- and 141-fold at 15 °C and 8 °C, respectively. 

The *O. antarictica* Cpn60/10 exhibited a higher in vitro refolding activity at lower temperatures compared with the *E. coli* Cpn60/10. In addition, the *E. coli* sHsps, IbpA/B were reported to be involved in resistance to copper-induced oxidative stress [14], and the cyanobacterial small heat-shock protein HspA was also found to play roles in enhancing the bacterial tolerance to oxidative stress and stabilization of the thylakoid membrane proteins [15]. This indicates that sHsps from different organisms could have diverse functions.

However, less is known about the archaeal sHsps, and the relevant studies are restricted to those from a few hyperthermophilic archaea, like the *Sulfolobus tokodaii* StHsp14.0 [16,17], the *Methanocaldococcus jannaschii* MjHsp16.5 [18], and the *Archaeoglobus fulgidus* Hsp20.2 [19]. Most studies on archaeal sHsps are focused on structural dynamics related functions. *Methanolobus psychrophilus* R15 is a rare cultured psychrophilic archaeon that grows optimally at 18 °C [20]. R15 possesses three sHsps paralogs, Hsp17.6, Hsp18.9, and Hsp20.2, which exhibit 58.5% amino acid sequence similarities, and all possess an α-crystallin domain (Figure S1). 

The three sHsps paralogs all increase transcriptions among the overall differential expressed genes in 4 °C- or 8 °C- compared with 18 °C-cultured *M. psychrophilus* R15. In particular, the gene encoding Hsp17.6 upregulated transcription by 11-fold at lower temperatures [21,22], suggesting that these sHsps may play a role in the cold adaptation of Archaea. Of the three, Hsp17.6 is the smallest but contains the most conserved α-crystallin domain; thus, it may possess the most related chaperone activities.

In this study, through the integration of biochemical and molecular approaches and evaluation of oxidant resistance of the sHsp heterologous expressed *E. coli*, we reported that Hsp17.6 from the psychrophilic archaeon *M. psychrophilius* R15 plays a role in the mitigation of protein oxidative inactivation and improvement of *E. coli* survivability in H_2_O_2_ and HClO, likely via oxidation of its aspartate residues to consume environmental oxidants.

## 2. Results 

### 2.1. The Small Heat Shock Proteins of M. psychrophilus Elevated the Stationary Cell Density of E. coli

*M. psychrophilus* possesses three sHsp paralogs, in which *Mpsy_2176* encodes a 17.6 kDa protein and was assigned as Hsp17.6; *Mpsy_0075* encodes an 18.9 kDa protein and was assigned as Hsp18.9; and *Mpsy_0869* encodes a 20.2 kDa protein and was assigned as Hsp20.2. All three possess the α-crystallin domain (Figure S1). 

To first test the canonical characteristics of the *M. psychrophilus* sHsps in alleviating the thermal aggregation of proteins, we heterologously expressed the three proteins with N-terminal 6×His and SUMO fusions, purified using a Ni-affinity column, and simultaneously removed the tag with SUMO protease ULP1 to obtain tag-free proteins. 

Hsp17.6, Hsp18.9, and Hsp20.2 were each mixed with either citrate synthase (CS) or luciferase at a 0−20:1 molar ratio. The three sHsps suppressing thermal aggregation of CS and luciferase were determined in dose-dependent manner based on monitoring the light scattering profiles at 360 nm of 45 °C-incubated protein mixtures (Figure S2). It is worth noting that Hsp17.6 exhibited much weaker activity compared with the other two, by showing a similar suppression effect at four-fold as that of Hsp18.9 and Hsp20.2 at two-fold to CS (Figure S2A), and a much weaker activity at as higher as 20-fold than that of Hsp18.9 and Hsp20.2 at two-fold to luciferase (Figure S2B). 

The *E. coli* strains that carry one of the three *M. psychrophilus* Hsps or IbpA/B achieved better growth at 22 °C, suggesting their roles in cold protection. Unexpectedly, the *E. coli* strains all obtained a higher stationary cell density compared with the vacant vector strain at 22, 37 and 45 °C (Figure S2C–E). In particular, expression of Hsp17.6 and Hsp18.9 also appeared to alleviate the 45 °C-cultured cell lysis in the stationary phase (Figure S2E). Given that more reactive oxygen species (ROS) would be accumulated in stationary cells [23,24], we hypothesize that the archaeal Hsp17.6 might have oxidation protective activity.

### 2.2. Hsp17.6 Overexpressed E. coli Gained Higher Survivability in Oxidants 

**Figure 1:**
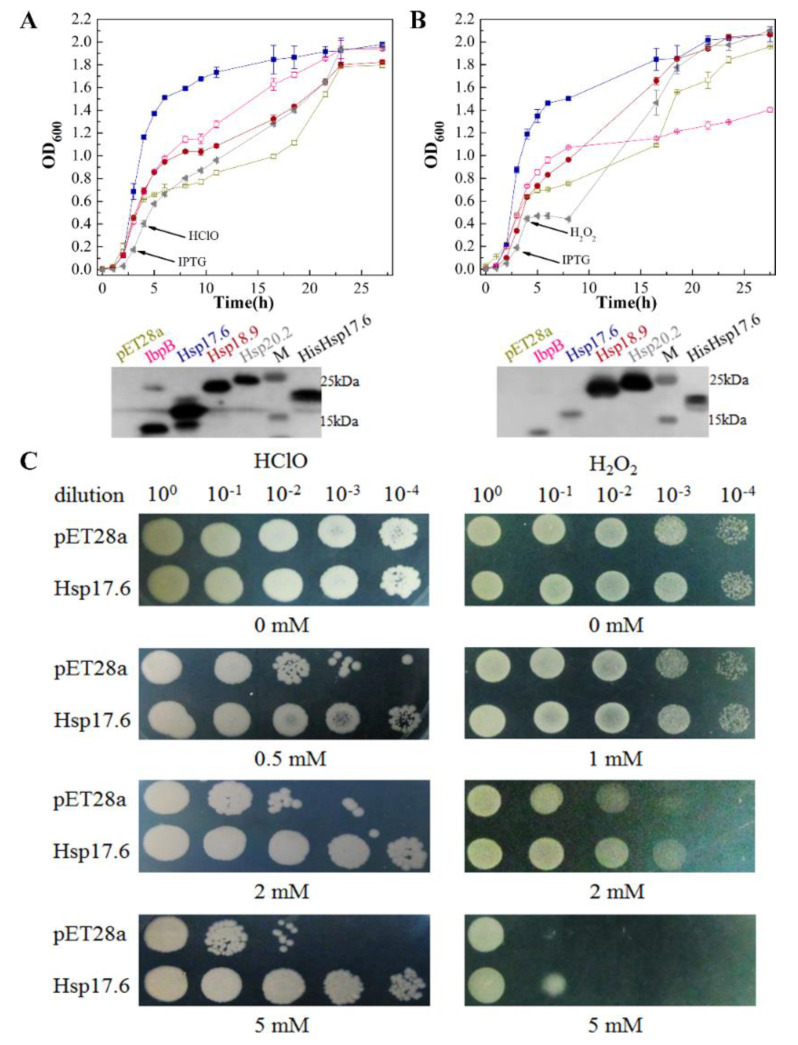
The *Methanolobus psychrophilus* Hsp17.6-expressing *Escherichia coli* exhibited enhanced growth and survivability in oxidants. The C-terminal His-tag fused Hsp17.6, Hsp18.9, Hsp20.2, and the *E. coli* IbpA/B were each cloned in the vector pET28a and expressed in *E. coli* JM109 (DE3), and the vacant vector was expressed as a control. All the strains were cultured in Luria-Bertani (LB) broth containing 50 μg/mL kanamycin at 37 °C, until the mid-exponential phase 0.1 mM IPTG was added to induce sHsps expression at the arrows indicated time. After another hour of incubation, 0.5 mM HClO (**A** upper panel) or 2 mM H_2_O_2_ (**B** upper panel) was added to the cultures. Growth was monitored via measurement of the OD_600_. ■, Hsp17.6; ●, Hsp18.9; ◀, Hsp20.2; ◯, IbpAB; and □, empty vector pET28a. The oxidant non-treated growths are shown in Figure S2 D. Experiments were assayed in triplicate, and the averages and standard deviations are shown. Using the His-tag antiserum, western blotting was used to assay the expression of the four proteins (lower panels of **A** and **B**) in 20 μg of total cell protein three hours after the addition of isopropyl-β-d-thiogalactoside (IPTG) and 0.2 μg purified Hsp17.6 carrying N terminal His-tag and additional 11 amino acids derived from the vector was included. Western blot signals were visualized by the Tanon-5200 Chemiluminescent Imaging System. (**C**) Strains carrying Hsp17.6 and the empty vector pET28a were 10-fold serially diluted and spotted to LB agar plates containing various concentrations of HClO (left panel) and H_2_O_2_ (right panel). Growths were recorded after overnight incubation at 37 °C.

To investigate if the archaeal sHsps have an oxidation protective activity, *E. coli* strains that carried the encoding genes of Hsp17.6, Hsp18.9, and Hsp20.2 were tested for oxidant resistance, and the *E. coli* IbpA/B-expressing strain was tested in parallel. After one-hour induction of Hsps expression by isopropyl-β-d-thiogalactoside (IPTG), 0.5 mM HClO or 2 mM H_2_O_2_ was added to the mid-exponential cultures. We found that, except for the strain expressing Hsp17.6, which continued to grow at a similar rate as non-treated, those carrying other sHsps and the empty vector paused growth for a few hours after the addition of oxidants and then slowly recovered (Figure 1A,B). This suggests that Hsp17.6 could possess an oxidation protective activity. Nevertheless, it must be cautious by using the heterologous host to assay the physiological functions of sHsps, as the heterologous expressed contents (about 1−3% total cellular proteins) may not be the same as in their native organisms. 

To confirm the oxidation protective activity of Hsp17.6, an exponential culture of *E. coli* expressing Hsp17.6 was 10-fold diluted and spotted on Luria-Bertani (LB) plates containing 0–5 mM of HClO or H_2_O_2_. Compared with the strain carrying the empty plasmid, which was inhibited by 0.5 mM HClO, the strain expressing Hsp17.6 survived in up to 5 mM HClO even at 10^−4^ dilution (Figure 1C left). Similarly, the Hsp17.6-expressing *E. coli* exhibited a higher tolerance to 2 and 5 mM H_2_O_2_ compared with the Hsp17.6-void strain (Figure 1C right); however, there was a less marked survival difference than with HClO. These results confirm the role of Hsp17.6 in assisting bacteria to resist oxidative stress.

### 2.3. Hsp17.6 Mitigated Protein Inactivation by Oxidants 

Given that oxidants, like H_2_O_2_ or HClO, primarily exert oxidative damage to proteins [23,25], we tested the *M. psychrophilus* Hsp17.6 in protection of oxidant-caused inactivation of enzyme proteins. Malate dehydrogenase (MDH) and superoxide dismutase (SOD), which have been conventionally used in the assessment of the thermal aggregation of proteins, were used as substrates. After assessing the concentrations of H_2_O_2_ and HClO to which MDH and SOD were sensitive, 0.2 and 1 mM of HClO and 10 and 1 mM of H_2_O_2_ were used to oxidize MDH and SOD, respectively. Purified Hsp17.6 protein was mixed with the enzymes at various molar ratios and then challenged with the oxidants, and the retained enzymatic activities were assayed within 20 or 30 min. 

**Figure 2:**
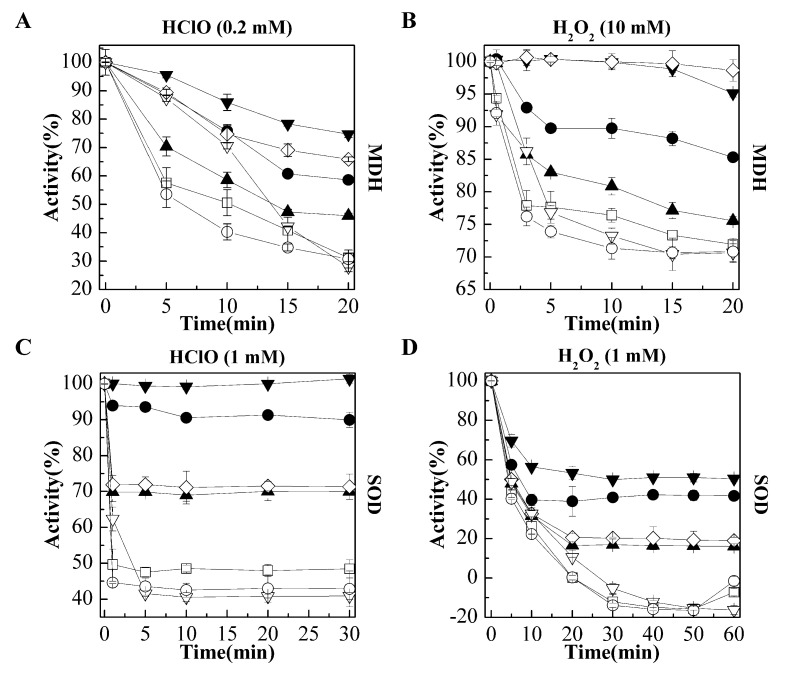
The addition of *M. psychrophilus* Hsp17.6 alleviated HClO or H_2_O_2_ oxidized inactivation of malate dehydrogenase (MDH) and superoxide dismutase (SOD). Hsp17.6 was mixed with 5.4 μM of MDH monomer (**A**,**B**) or 7.8 μM of SOD monomer (**C**,**D**) at the indicated molecular ratios, and the two paralogs Hsp18.9 and Hsp20.2 were also mixed with MDH and SOD, respectively. In addition, MDH and SOD were reciprocally used as a negative control. After addition of the indicated concentrations of HClO or H_2_O_2_ into the protein mixtures, the corresponding enzyme activities were assayed at different time points. The residual activity percentages were calculated by reference to those at 0 min. The experiments were carried out in triplicate, and the averages and standard deviations are shown. (**A**). MDH was co-incubated with 0 (□), 10.8 μM (▲), 21.6 μM (●), and 43.2 μM (▼) of Hsp17.6; and 21.6 μM of Hsp18.9 (◊) or Hsp20.2 (∇), or SOD (◯). (**B**). MDH was co-incubated with 0 (□), 0.54 μM (▲), 2.7 μM (●), and 10.8 μM (▼) of Hsp17.6; and 21.6 μM of Hsp18.9 (◊) or Hsp20.2 (∇), or SOD (◯). (**C**). SOD was co-incubated with 0 (□), 15.6 μM (▲), 31.2 μM (●), and 62.4 μM (▼) of Hsp17.6; and 31.2 μM of Hsp18.9 (◊) or Hsp20.2 (∇), or MDH (◯). (**D**). SOD was co-incubated with 0 (□), 15.6 μM (▲), 31.2 μM (●), and 62.4 μM (▼) Hsp17.6; and 31.2 μM of Hsp18.9 (◊) or Hsp20.2 (∇), or MDH (◯).

In parallel, Hsp18.9 and Hsp20.2 were tested for the oxidation protective activity, and SOD and MDH were also reciprocally added as controls. We determined that the addition of Hsp17.6 alleviated the oxidative inactivation of the two enzymes in a dose-related manner, as only 57% of the MDH activity remained in the first 5 min with the 0.2 mM HClO challenge; however, ≥90% activity was assayed in the presence of Hsp17.6 at ratios of 4−8:1 to MDH (Figure 2A). Hsp17.6 showed a higher protection on 10 mM H_2_O_2_ treated MDH as well, as its addition at 2:1 almost completely recovered the MDH activity (Figure 2B). 

SOD appeared to be more sensitive to the two oxidants; only 50% of the activity was retained within 1 and 5 min after the addition of 1 mM of HClO or H_2_O_2_, respectively, whereas, 70% and 94% of the SOD activities were assayed within the first min when Hsp17.6 was present at 2:1 and 4:1, respectively (Figure 2C). Similarly, only no SOD activity in 1 mM H_2_O_2_ was assayed within 20 min, while 20–58% activity was retained in the presence of Hsp17.6 (Figure 2D). 

The addition of four-fold Hsp18.9 also recovered the HClO- and H_2_O_2_-treated SOD activity, and the H_2_O_2_-treated MDH activity similarly as addition of two-fold Hsp17.6 (Figure 2B–D), and retained higher HClO-treated MDH activity than addition of four-fold Hsp17.6 (Figure 2A). While, Hsp20.2 exhibited weak effects, so presenting different oxidation protective activities among the three paralogs. The reciprocal addition of four-fold MDH and SOD did not alleviate the oxidative inactivation of each other from HClO or H_2_O_2_, thus precluding the possibility that the observed Hsp17.6 oxidation protective activity was from the protein crowding effect. 

In addition, HyPer, an H_2_O_2_-specific reporter fluorescence protein, was used as substrate to assay Hsp17.6 in alleviating the oxidative inactivation of proteins. The HyPer protein was constructed by Belousov et al. [26] by inserting the regulatory domain of the *E. coli* H_2_O_2_-sensing protein OxyR into the fluorescent protein cpYFP. When H_2_O_2_ oxidizes Cys199 and Cys208 to form a disulfide bond, HyPer emits green fluorescence. With the addition of 1 mM H_2_O_2_ to 450 nM HyPer, elevated fluorescence was detected; and the addition of 1.8 μM Hsp17.6 or Hsp18.9 to the mixture reduced the H_2_O_2_-induced HyPer fluorescence increasing (Figure S3). Whereas, no such effect was assayed for Hsp20.2. This provides further evidence that Hsp17.6 and Hsp18.9, but not Hsp20.2, are capable of alleviating the oxidation inactivation of proteins. 

### 2.4. Hsp17.6 Appeared to Alleviate the H_2_O_2_-Oxidized Disulfide Linking and the HClO-Induced Degradation of Proteins

**Figure 3:**
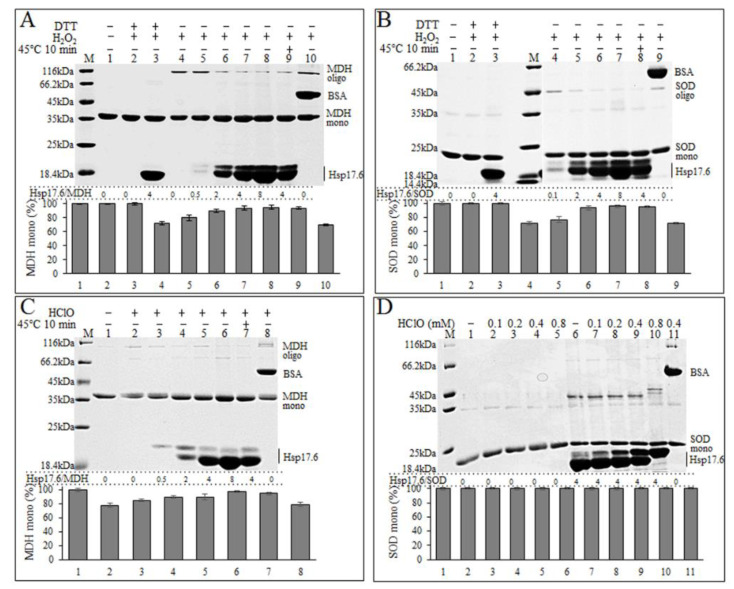
The presence of Hsp17.6 reduced the oxidant-induced disulfide linking protein contents. Protein mixtures of MDH (**A**) or SOD (**B**) with various molecular ratios of Hsp17.6 were preheated at 45 °C for 10 min before 1 mM H_2_O_2_ treatment for 30 min, and then reduced with 2 mM DTT for 30 min. Mixtures of MDH with various ratios of Hsp17.6 were also treated for 30 min with 0.4 mM HClO (**C**), and SOD with four-fold Hsp17.6 was treated for 30 min with 0–0.8 mM HClO (**D**). All the treatments were performed at 37 °C unless otherwise indicated. The treated protein mixtures were analyzed on non-reducing sodium dodecyl sulfate polyacrylamide gel electrophoresis (SDS-PAGE) (12%) and stained by Coomassie blue. Beneath the gels shows the molecular ratios of Hsp17.6 to the substrate proteins and the percentages of the substrate monomer band intensities referenced to the non-treated ones. The bovine serum albumin (BSA) at 0.5:1 molecular ratio to MDH or SOD was included as a control. Protein markers and the tested proteins are shown at the left and right of the gels, respectively. The MDH and SOD monomer intensities were scanned and calculated using ImageJ, and the averages and standard deviations from triplicate experiments are shown.

To probe the mechanisms of Hsp17.6 in alleviation of the oxidative inactivation of proteins, we, using non-reducing sodium dodecyl sulfate polyacrylamide gel electrophoresis (SDS-PAGE), analyzed the oxidative status of oxidant-treated MDH and SOD in the presence or absence of Hsp17.6. As shown in Figure 3, a 30 min-treatment with 1 mM H_2_O_2_ caused certain proportions of MDH and SOD to migrate slower with increased apparent molecular weights (lanes 4 of Figure 3A,B); however, the larger protein bands disappeared with addition of 2 mM DTT (lanes 2 of Figure 3A,B). This indicates that H_2_O_2_ oxidation caused disulfide-linked oligomerization of MDH or dimerization of SOD based on the theoretical molecule weights of the MDH monomer (32 kDa) and SOD monomer (21 kDa). With the addition of Hsp17.6 at molar ratios of 2−8:1, the oxidized MDH oligomer (Figure 3A, lanes 2−8), and the SOD dimer contents (Figure 3B, lanes 5–7) decreased, and the monomers increased from about 70% to 95%. 

Preheat treatment at 45 °C did not change the effect of Hsp17.6 in reducing the oxidized oligomer contents, and the tested bovine serum albumin (BSA) did not show protection on MDH (Figure 3A, lane 10) and SOD oxidation (Figure 3B, lane 9). It is worthy of noting that a relatively larger protein band of Hsp17.6 occurred in the H_2_O_2_-treated mixtures (Figure 3). This assumes that Hsp17.6 could protect the substrate proteins from oxidant-caused disulfide-bond formation by itself being oxidized. 

Unlike H_2_O_2_ oxidation, which led to disulfide linkages in proteins, 0.4 mM HClO caused oxidative degradation of MDH (Figure 3C, lane 2), and a severe degradation occurred in 0.8 mM HClO (Figure S4). HClO-caused MDH degradation was gradually mitigated by the addition of 0.5−8:1 Hsp17.6 (Figure 3C, lane 3−6). In contrast, HClO appeared to cause neither the protein size changes nor the degradation of SOD; rather, the addition of Hsp17.6 induced a slower migrating protein band presumably of SOD (Figure 3D). 

It is worth noting that in the presence of higher oxidant concentrations, like 0.8 mM HClO, Hsp17.6 was fully oxidized, however the MDH oligomer contents were not further reduced (Figure S4). This indicates the limitation of Hsp17.6 as an antioxidant. Considering that HClO treatment markedly reduced the SOD activity (Figure 2C), HClO oxidation resultant disulfide linkages of proteins could be among the multiple oxidative consequences, such as the irreversible oxidation to sulfonic acid or sulfenic acid of the thiol groups and protein carbonylation. 

### 2.5. Mass Spectrometry Detected the Oxidized Amino Acid Residues of Hsp17.6 

**Figure 4:**
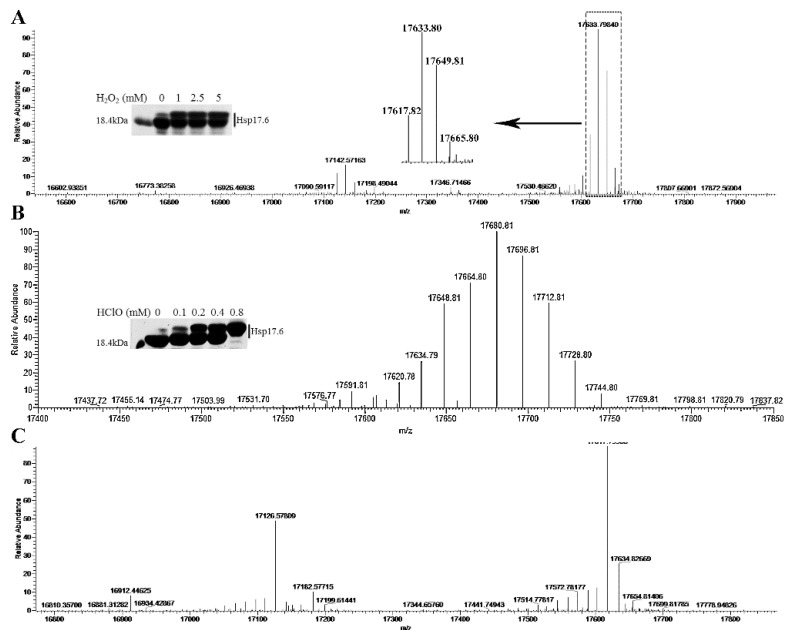
Identification of the molecular weights of Hsp17.6 after treatment with H_2_O_2_ or HClO using high performance liquid chromatography-mass spectrometry (LC-MS). Hsp17.6 protein treated with 5 mM H_2_O_2_ (**A**) or 0.4 mM HClO (**B**) or without treatment (**C**) subjected to orbitrap fusion for analysis. Hsp17.6 protein treated for 30 min with 0–5 mM of H_2_O_2_ (**A**, insert) or 0–0.8 mM of HClO (**B**, insert) and separated on non-reducing SDS-PAGE.

Unlike thioredoxin or other redox proteins that reduce H_2_O_2_- or HClO- oxidized proteins via oxidation of the thiol groups of cysteine, the Hsp17.6 protein does not contain cysteine residues. To obtain insights into the molecular changes of Hsp17.6 in alleviating the oxidation of other proteins, it was treated by 0–5 mM H_2_O_2_ and 0−0.8 mM HClO. Compared with the non-treated one, the H_2_O_2_- and HClO-treated Hsp17.6 proteins were run as two bands on non-reducing SDS-PAGE (Figure 4A,B inserts) similar as the oxidant-treated protein mixtures with MDH and SOD. 

Next, 5 mM H_2_O_2_- and 0.4 mM HClO-treated Hsp17.6 proteins were subjected to high performance liquid chromatography-mass spectrometry (LC-MS) to determine the molecular weights. Compared with the non-treated Hsp17.6 (Figure 4C), the H_2_O_2_-treated proteins increased by about 16, 32, and 48 daltons (Figure 4A), and the HClO-treated proteins increased by about 32, 48, 64, 80, and 96 daltons (Figure 4B). This indicates that oxygen atoms may be introduced into Hsp17.6 upon oxidant treatment.

The oxidization enlarged Hsp17.6 protein was tryptase hydrolyzed and subjected to liquid chromatography-tandem mass spectrometry (LC-MS/MS) identification of the oxidized residues. The non-oxidized smaller band was analyzed in parallel. Compared with the relative abundances of the oxidized peptide fragments in H_2_O_2_- and HClO-treated vs. non-treated Hsp17.6, LC-MS/MS identified methionine (M), aspartate (D), asparagine (N), and lysine (K) as the main residues with oxygen atoms introduced (Figure S5). As shown in Table S1, in H_2_O_2_-treated Hsp17.6, 74% M16, 100% M25, and 69% M103 were introduced with an extra oxygen atom compared with 32%, 47%, and 48% oxygen introduction in the non-treated protein. Similarly, 1.5−5-fold higher oxygen introduction was found in D44, D46, D53, D66, and D132 in the H_2_O_2_-treated vs. non-treated Hsp17.6. Collectively, mass spectrometry identified that, except for methionine residues, the aspartate and asparagine residues of Hsp17.6 were readily oxidized by H_2_O_2_ and HClO. This indicates that Hsp17.6 might exert a protective role in protein oxidation via consumption of the oxidants that the protein encounters.

In addition, LC-MS/MS also identified similar extra oxygen atoms in HClO-oxidized MDH protein (Table S1), which was sliced from the non-reducing SDS-PAGE (Figure 3C, lane 2); while reduced abundances of oxygen introduction in MDH were found in the presence of Hsp17.6 (Table S1; Figure 3C, lane 5). This result confirmed the protective activity of Hsp17.6.

### 2.6. Substitution of Aspartate Residues Reduced the Protection of Hsp17.6 on Protein Oxidative Inactivation

**Figure 5:**
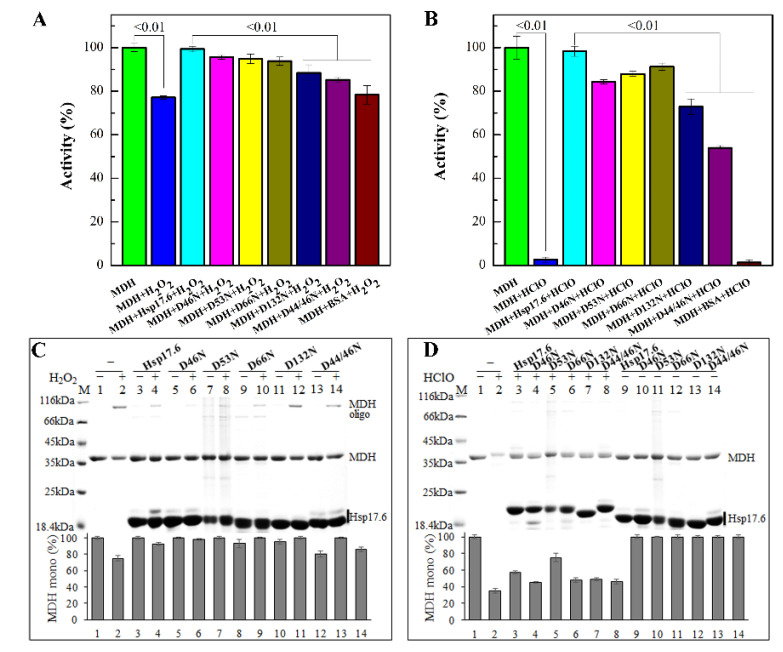
Substitution of certain aspartate residues reduced the Hsp17.6 effect in alleviating the oxidative inactivation of MDH. MDH was mixed with each of the wild-type and the aspartate mutants of Hsp17.6 at 2:1 (**A**) or 4:1 (**B**), and the activities in 10 mM H_2_O_2_ (**A**), or 0.4 mM HClO (**B**) were assayed using the same approach as in Figure 2. The averages and standard deviations of triplicate experiments are shown. Difference significances (indicated at the diagram top) of the oxidant-treated MDH enzymatic activities in the presence of Hsp17.6 wild-type and residue mutants were statistically analyzed by the ANOVA and Tukey multiple comparison test. Non-reducing SDS-PAGE was used to analyze the oxidation states of H_2_O_2_- (**C**) and HClO- (**D**) treated MDH, and Hsp17.6 and its mutants. Oxidant treatments were performed at 37 °C for 60 min. The Non-reducing SDS-PAGE was Coomassie blue stained. Protein markers and the tested proteins are shown at the left and right of the gels, respectively. MDH monomer band intensities were scanned and calculated using ImageJ, and the averages of and the standard deviations from triplicate experiments are shown.

Given that the Hsp17.6 asparagine residues were less sensitive to H_2_O_2_ and HClO oxidization than aspartate (Table S1), we constructed an asparagine substitution on D44, D46, D53, D66, and D132 and a double mutation of D44 and D46. The six Hsp17.6 protein mutants were each overexpressed in *E. coli.* Except for D44N, the remaining five were purified as soluble proteins, and were assayed for alleviating MDH oxidation by H_2_O_2_ and HClO. We determined that compared to almost 100% MDH activities assayed in the presence of wild-type Hsp17.6, 85–95% activities in 10 mM H_2_O_2_ (Figure 5A) and 54–91% activities in 0.4 mM HClO (Figure 5B) were found when the five mutants were added. Mutants of D132N and D44N/D46N caused a significant loss to the protective activity.

Assaying these protein mixtures on non-reducing SDS-PAGE, we found that compared with the wild-type Hsp17.6 addition, relatively higher contents of H_2_O_2_-induced MDH oligomers were observed when added with the mutants of D132N and D44N/D46N (Figure 5C); meanwhile, the larger band in the five H_2_O_2_-treated mutants almost disappeared. This result suggests that the mutated residues could be the oxidized targets of Hsp17.6. 

Compared with the wild-type Hsp17.6 in mitigating HClO-induced MDH degradation, the mutants D46N, D66N, D132N, and D44N/D46N only exhibited weak effects. These residues appeared being more oxidized in the presence of HClO as indicated by mass spectrometry analysis, suggesting that they could be the oxidation targets of Hsp17.6; however, the oxidized larger band of Hsp17.6 only disappeared in D132N (Figure 5D). These data suggest that mutated aspartate residues, like D132, could be the general oxidized targets of Hsp17.6 by the two oxidants and, together with the other aspartate residues, contribute to the antioxidative role of Hsp17.6. 

### 2.7. The Hsp17.6 Aspartate Residue Mutants Reduced Attenuating Oxidant-Suppressed E. coli Growth 

**Figure 6:**
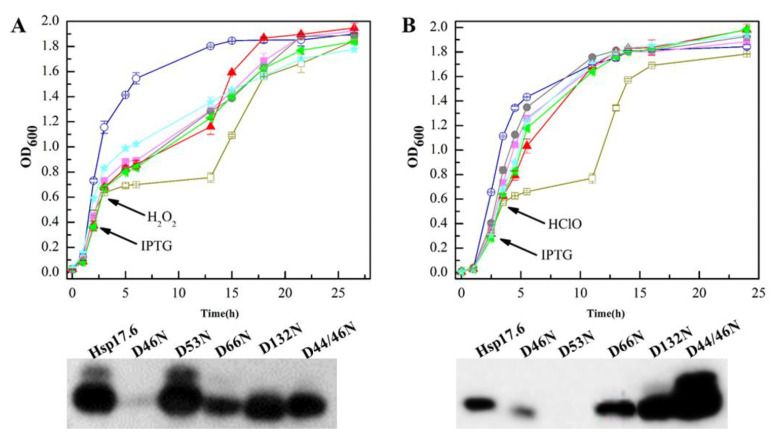
The Hsp17.6 aspartate mutants reduced the effect of attenuating oxidant-suppressed growth of *E. coli*. Five aspartate substitution mutants, D46N (■), D66N (●), D53N (◄), D132N (★), and D44N/46N (▲), were each introduced into *E. coli* JM109(DE3) via the vector pET28a. The wild-type Hsp17.6 (**◯**) and empty pET28a (**□**) were expressed as controls. The *E. coli* strains were grown in LB broth, and, after 2–2.5 h-incubation, when the OD_600_ reached 0.6–0.8, 0.1 mM IPTG was added to induce the expression of the Hsp17.6 mutants. After 1 h of induction, 2 mM H_2_O_2_ (**A** upper panel) or 0.5 mM HClO (**B** upper panel) were added. Growth was monitored by measurement of the OD_600_. The protein expression was assayed 3 h after the addition of IPTG by western blot using the His-tag antiserum (lower panels of A and B) and visualized by the Tanon-5200 Chemiluminescent Imaging System. Triplicate cultures of each mutant were assayed, and the averages and standard deviations are shown.

To further verify the role of the mutated aspartate residues in the Hsp17.6 oxidation protection, the five mutants were introduced into *E. coli* JM109 (DE3) via the vector pET28a, and the wild-type Hsp17.6 and empty pET28a expressing strains were included as controls. Growths of 0.5 mM HClO- or 2 mM H_2_O_2_-treated strains were followed. Expect for D46N and D53N, which were poorly expressed in the H_2_O_2_- and HClO-treated cultures, respectively, most of the Hsp17.6 mutant proteins were expressed (Figure 6 lower panels). 

However, compared with the wild-type Hsp17.6-expressing *E. coli*, those carrying the mutants all exhibited marked reduced growth when treated with H_2_O_2_ (Figure 6A upper panel), and the growths were recovered until the stationary phase presumably due to the *E. coli* catalase removing the added H_2_O_2_. The mutant-expressing *E. coli* did not exhibit increased sensitivity to HClO (Figure 6B upper panel). These results confirm that the mutated aspartate residues have contributions to Hsp17.6 in alleviating the H_2_O_2_-, but not HClO-oxidized inactivation of proteins and in elevating the bacterial growth under oxidative stress. 

## 3. Discussion

Small heat shock proteins are well known in mitigation of protein aggregation at high temperatures. This work reports that the archaeal sHsp Hsp17.6 and the paralog Hsp18.9 from *M. psychrophilus*, a psychrophilic methanogenic archaeon, alleviated the protein oxidative inactivation and oxidative stress of *E. coli* imposed by H_2_O_2_ and HClO, thus, exhibiting an oxidation protection function. 

Some of the Hsp17.6 aspartate residues appeared susceptive to oxidants, and their oxidations could consume certain oxidants and reduce the oxidant insult to proteins SOD and MDH, and reduce oxidized disulfide-linked oligomers of them. Hsp17.6 orthologs are distributed among some archaea and bacteria, and the predicted oxidant-susceptive aspartate residues are conserved in the orthologs (Figure S6); therefore, the reported oxidation protective role of the archaeal sHsps in this work could be widely applicable.

Higher dissolved oxygen contents could occur in cold water systems [27]; therefore, the inhabiting psychrophilic or psychrotolerant organisms would likely have evolved more approaches to cope with the increased oxidative stress compared with mesophiles. Previously, we found that *M. psychrophilus* R15 was able to survive up to 0.8 mM paraquat, a redox-cycling drug, and in comparison, a mesophilic methanogen only tolerated as low as 0.05 mM paraquat [21]. 

In addition to oxidant-removing systems, like the anaerobe-specific superoxide reductase (SOR) and aerobe-typical superoxide dismutase-catalase, the sHsps with oxidation protective roles could also contribute to the higher survivability of *M. psychrophilus* in reactive oxygen species (ROS). The three *M. psychrophilus* sHsps all fell in the top 10 over-representative expressed gene categories in lower temperatures [22], implying that they could play roles in assisting the archaeon to survive in either cold or higher oxidative stress in cold. Nevertheless, the physiological functions of Hsp17.6 in *M. psychrophilus* remain to be elucidated until a genetic manipulation system is developed in the archaeon. 

Bacterial sHsps were also reported in protection of bacteria from oxidative stress. The *E. coli* IbpA/B proteins were determined to protect the bacterium against copper-ion-induced oxidative stress, and in the presence of IbpA/B lower levels of the carbonylated proteins were found in copper-ion-treated *E. coli* [14]. Sakthivel et al. [15] also reported that HspA endowed *Synechococcus* with higher survivability in H_2_O_2_, through specifically suppressing H_2_O_2_ bleaching phycocyanin via a direct interaction with phycobilisome. However, the molecular details regarding how the two bacterial sHsps protect protein oxidation either from copper-induced ROS or directly from H_2_O_2_ remain to be determined. 

The archaeal Hsp17.6 could be sensitive to oxidants, and an oxidized larger band occurred even during protein preparation (Figure 4A). Though the mechanism details have not been elucidated yet, this work determined that the archaeal Hsp17.6 protected the oxidative inactivation of proteins most likely through aspartate residue oxidation to consume the oxidants H_2_O_2_ and HClO. Conventionally, it is believed that cysteine and methionine are the two residues most vulnerable to H_2_O_2_ oxidation, and H_2_O_2_ did induce disulfide-linked MDH and SOD oligomers as observed in this study (Figure 3A,B). 

Low concentrations of HClO could rapidly induce the oxidized protein unfolding in vitro and cause irreversible protein aggregation in vivo [28]. The bacterial chaperone Hsp33 is activated when its cysteine is oxidized by oxidants, such as H_2_O_2_ or HClO, and inhibits the aggregation of other proteins [28]. Meanwhile, oxidation of the Hsp33 Zn^2+^ coordinated cysteine residues alter the protein conformation due to the Zn^2+^ lost [29]. The Hsp17.6 aspartate residues appear to be readily oxidized by introduction of oxygen atoms into the protein; however, the oxygen modified aspartate groups remain to be identified.

Through amino acid sequence comparison among the Hsp17.6 orthologs from archaeal and bacterial species, we found that certain mutated aspartate residues in this study, in particular D53, D66, and D132, were conserved (Figure S6), while some of them were absent in Hsp20.2 (Figure S1), the paralog did not show the same oxidation protective activity (Figure 1 and Figure 2). These results suggest that the aspartate residues could play a key role in the oxidative protection of the sHsps.

## 4. Materials and Methods

### 4.1. Materials

The citrate synthase and mitochondrial malate dehydrogenase from porcine heart, and bovine serum albumin and the superoxide dismutase from *Escherichia coli* cell lyophilized powder were all purchased from Sigma-Aldrich company (St. Louis, MO, USA). Luciferase was purchased from Promega company (Madison, WI, USA). Sodium hypochlorite and hydrogen peroxide were purchased from Macklin (Shanghai, China). Gibson Assembly^®^ Master Mix was purchased from NEB (Ipswich, MA, USA). The ubiquitin-like-protease 1 that is the C-terminal fragment (403−621 amino acids) of the yeast small ubiquitin-like modifier protease (SUMO protease ULP1, Gene ID: 856087) was fused with a His6-tag at the N-terminal, and heterogenous expressed in *E. coli* and purified.

### 4.2. Growth Experiments

The archaeal Hsp17.6, Hsp18.9 and Hsp20.2, and the *E. coli* IbpA/B were each cloned into the plasmid pET28a and transformed into *E. coli* JM109(DE3). A single colony of each transformant was cultured in Luria-Bertani (LB) broth at 37 °C overnight. After adjusting cell numbers, 1% (*v/v*) culture was inoculated into 50 mL LB medium containing 50 μg/mL kanamycin that was contained in 100 mL-Erlenmeyer flask and incubated at 37 °C with shaking at 200 rpm. Until 2–3 h-incubation when OD_600_ reached 0.4 − 0.6, 0.1 mM isopropyl-β-d-thiogalactoside (IPTG) was added to induce protein expression of the Hsps. After another hour induction, triplicate cultures were respectively incubated at 22 °C, 37 °C and 45 °C, and the other replicate cultures were added with 2 mM H_2_O_2_ or 0.5 mM HClO for oxidant-treated growth assay. OD_600_ was measured in an interval of one or two hours during the growth.

For oxidant survival experiments, *E. coli* strains that are expressed with Hsp17.6 or empty vector were cultured as above. After 2.5 h-IPTG induction, cultures are adjusted to the same OD_600_ values of 1.0 and each 6 μL of 10-fold diluted cultures were spotted on 1.5% LB agar plates that contain 50 μg/mL kanamycin, 0.1 mM IPTG and 0–5 mM of H_2_O_2_ or HClO as indicated. After overnight incubation at 37 °C, growths were recorded.

### 4.3. Construction of Hsp17.6 Residue Substitution Mutants

The genomic DNA of *Methanolobus psychrophilus* R15 was extracted using Bacteria Genome DNA extraction kit (OMEGA, New York, USA) and used as PCR template. Primers were designed according to the genome sequence [21] and synthesized by Sangon Company (Shanghai, China). The Hsp17.6 gene (*Mpy_2176*) was amplified using the primer pair listed in Table S2, and cloned into pET28a. The residue substitution mutants were constructed through PCR amplification of pET28a-hisSUMO-Hsp17.6 or pET28a-Hsp17.6his using primers containing mutated triple-base with site-directed gene mutagenesis kit (Beyotime Biotechnology Company, Shanghai, China). The constructs were transformed into *E. coli* DH5α, and correct transformants were selected on LB plates containing 50 μg/mL kanamycin and verified by PCR and sequencing.

### 4.4. Protein Expression and Purification

The archaeal Hsp17.6, Hsp18.9 and Hsp20.2 genes were each fused a 6× His plus SUMO (small ubiquitin-like modifier) tag at the N-terminals the N-terminal via Gibson assembly. The constructs were each cloned into pET28a, and after DNA sequencing examination, correct plasmids were transformed into *E. coli* BL21(DE3). A single *E. coli* colony was inoculated into LB broth with 50 μg/mL kanamycin and 200 rpm-shaking cultured at 37 °C. When OD_600_ reached 0.4, one mM IPTG was added to induce expression of the sHsps, and after another 6 h-incubation, cells were harvested and resuspended in binding buffer (20 mM sodium phosphate, pH 8.0, 500 mM NaCl, 20 mM imidazole). Cells were lysed by ultrasonication using JY92-2D ultrasonic cell crusher (Xinzhi Biotechnology Co. LTD, Ningbo, China) at 400 W for 30 min, and cell lysates were eluted through His Trap HP (GE Healthcare) to capture sHsps. The purified proteins were then removed the SUMO tag with His-tagged SUMO protease ubiquitin-like protease 1 via dialysis against the binding buffer, and then loaded to the His Trap HP again. The eluate was collected and further purified via superdex200 size exclusion chromatography for 30 mL elution of 0.5 mL/min in a buffer containing 20 mM Tris, 150mM NaCl and 5% glycerol. The sHsp preparations were concentrated and stored at −80 °C.

### 4.5. Enzymatic Assays on Oxidants Treated Proteins

A final concentration of 5.4 μM MDH was mixed with each 0 − 43.2 μM of Hsp17.6, and 21.6 μM of Hsp18.9 or Hsp20.2, or SOD in 0.1 M potassium phosphate buffer (pH7.5). After treated with 0−10 mM H_2_O_2_ or 0−0.4 mM HClO for a given time periods, MDH activity was assayed using oxaloacetate reduction method [30]. Briefly, 5 μL of the oxidant treated protein mixture were added to a final volume of 1 mL potassium phosphate buffer containing 0.2 mM NADH and 5 mM oxaloacetate. Absorbance of 340 nm was monitored by a spectrophotometer (UV-2550, SHIMADZU, Kyoto, Japan) at 25 °C at 30 s-interval for 2 min. Measurements were done for triplicate samples.

A final concentration of 7.8 μM SOD was mixed with 0−62.4 μM Hsp17.6, and 31.2 μM of Hsp18.9 or Hsp20.2, or MDH in a Tris-EDTA buffer (50 mM Tris, 100 mM EDTA, pH8.0). After treated with 0−1 mM H_2_O_2_ or 0−1 mM HClO for a given time period, SOD activity was measured by the pyrogallol (1,2,3-trihydroxybenzene) autoxidation method [31]. Briefly, 120 μL of the oxidant treated protein mixture were added to a final volume of 600 μL Tris-EDTA buffer containing 10 mM pyrogallol. Absorbance at 325 nm was monitored by a UV-2550 spectrophotometer at 25 °C at an interval of 30 s for 5 min. Triplicate samples were assayed.

### 4.6. Light Scattering Assay

Light scattering assay was used to assess the roles of sHsps in reducing protein thermal aggregation. Citrate synthase (CS) and luciferase were used as the substrate proteins. CS monomer in 600 nM was incubated with 0−12 μM of Hsp17.6, Hsp18.9 and Hsp20.2 in 40 mM HEPES-KOH, pH7.5. While, 1 μM of luciferase was incubated with 0−20 μM of each of sHsps in 20 mM tris, 150 mM NaCl, pH 8.0. The protein mixtures were incubated at 45 °C and the light scattering at 360 nm was continuously monitored within 60 min by Beckman Coulter DU800 spectrophotometer.

### 4.7. Mass Spectrometry Analysis

For molecular weight determination of Hsp17.6 that is oxidized by 5 mM H_2_O_2_ or 0.4 mM HClO in a Tris-HCl buffer (20 mM Tris, 150 mM NaCl, 5% glycerol, pH 8.0) were analyzed by RP-HPLC-C18-MS using orbitrap fusion (Thermo Fisher Scientific, Massachusetts, MA, USA) after desalting by a Zip Tip_C18_ pipette tip (Millipore, Burlington, MA, USA). The non-treated Hsp17.6 protein was included as the reference. Protein solutions were desalted using Millipore Zip Tip pipette tips containing C18 reversed-phase media according to the procedure in user guide. Briefly, the tips were wetted with 100% acetonitrile (ACN) and equilibrated with 0.1% TFA in Milli-Q grade water. Then protein solutions were loaded on the tips and washed with 0.1% TFA in Milli-Q grade water to remove the salt. Finally, proteins were eluted using 0.1% TFA/50% ACN and completely dried using Speed-vac. The dried protein preparations were dissolved in 0.1% formic acid (FA) and analyzed with an EASY-nLC 1000 interfaced via a Nanospray Flex ion source to Orbitrap Fusion Tribrid mass spectrometer (Thermo Fisher Scientific). In details, protein samples were loaded onto a trap column (C18, 3 μm particles, 100 μm inner diameter (ID), 2 cm length, Dr. Maisch GmbH) and separated using an analytical column (C18, 1.9 μm particles, 150 μm ID, 15 cm length, Dr. Maisch GmbH) at a flow rate of 400 nL/min with a 30 min LC gradient composed of Solvent A (0.1% formic acid (*v/v*)) and Solvent B (acetonitrile, 0.1% formic acid (*v/v*)) and a gradient elution starting at 20% to 60% B for 25 min, from 60–90% B for 5 min. MS spectra were acquired by orbitrap detector with a resolution of 120,000 and the individual precursor windows were summed across the relevant retention time period where the intact protein elute and then deconvoluted using Xtract tool within Xcalibur Qual Browser.

Protein oxidation status was assayed by LC/MS-MS. The oxidized protein mixtures were separated on a non-reducing SDS-PAGE (12%) and stained with Coomassie blue R-250. Each protein band was cropped and after twice washing with MS-grade water directly alkylated with 55 mM iodoacetamide (IAM) for 1 h at 37 °C in the dark. Then, the proteins were in-gel digested overnight using sequencing-grade modified trypsin (Promega, Fitchburg, WI, USA) in 50 mM NH_4_HCO_3_ (pH 8.0) at 37 °C. LC-MS/MS analysis was implemented with the Easy-nLC integrated nano-HPLC system (Proxeon, Odense, Denmark) and Q-Extractive mass spectrometer (Thermo, Waltham, Massachusetts, MA, USA) as described previously [32]. MS/MS spectra were searched against the forward and reverse Hsp17.6 protein sequence (Protein ID: AFV24380) in the NCBI database using the SEQUEST search engine of Proteome Discoverer software (v1.4). The precursor ion mass tolerance was 20 ppm for all MS acquired in an Orbitrap mass analyzer and fragment ion mass tolerance was 0.02 Da for all MS/MS spectra. The search criteria were employed as follows: full tryptic specificity was required; two missed cleavages were allowed; oxidation was used as a variable modification; the false discovery rate (FDR) was set to 0.01. Triple experiments were performed in parallel.

### 4.8. Non-Reducing SDS-PAGE Analysis on Oxidized Proteins

MDH in the presence or absence of Hsp17.6 in 15 μL PBS (137 mM NaCl, 2.7 mM KCl, 10 mM Na_2_HPO_4_, 2 mM KH_2_PO_4_, pH7.4), was 30 min-treated by 1 mM H_2_O_2_ or 0–0.8 mM HClO at 37 °C, and then added to a 4-fold diluted non-reducing SDS loading buffer (0.2 M Tris-HCl, 40% glycerol, 8% SDS, 0.4% Bromphenol blue, pH 6.8). The protein buffer was loaded on 12% SDS-PAGE gel, and electrophoresed at 150 volts for 1 h, and stained by Coomassie blue R-250. The intensities of the target bands were quantified using ImageJ [33].

### 4.9. Western Blot

A total of 10 mL of cultures of the sHsps expressed *E. coli* strains was harvested by centrifuge at 8000 g for 5 min, resuspended in 400 μL PBS and lysed by ultrasonication at 240 W with a cycle of 3 s sonication and 3 s pause for 10 min on ice. After being quantified by the Pierce BCA Protein Assay Kit (Thermo Fisher Scientific), total proteins of 20 μg was electrophoresed on SDS-PAGE under 150 V for 1 h, and the separated proteins were then transferred to a 0.1-mm nitrocellulose filter membrane (Easybio, Beijing, China) under 380 mA for 1 h. The membrane was blocked for 1 h at room temperature using 20 mL blocking buffer (50 g defatted milk powder, 2.42 g Tris, 8.0 g NaCl in 1 L, pH 7.6), and incubated with 1/5000 dilutions of Anti-His-Tag(2A8) mAb (HRP Conjugated) antibody (Abmart, Shanghai, China). The membrane was washed three times with Tris-buffered saline Tween buffer (2.42 g Tris, 8.0 g NaCl, 1mL Tween 20 in 1 L, pH 7.6), and signals were imaged via a Tanon-5200 Chemiluminescent Imaging System (Tanon Science & Technology, Shanghai, China).

## Data Availability

The data that support the findings of this study are available from the corresponding author upon reasonable request.

## Supplementary Materials

Supplementary materials can be found at https://www.mdpi.com/1422-0067/22/5/2591/s1.
