# Supplementary material for: The Archaeal Small Heat Shock Protein Hsp17.6 Protects Proteins from Oxidative Inactivation
Source: Int J Mol Sci. 2021 Mar 4;22(5):2591. doi: 10.3390/ijms22052591 (PMC7961418; doi:10.3390/ijms22052591)
Supplement: Supplementary file 1 [file ijms-22-02591-s001.pdf]

Supplementary materials

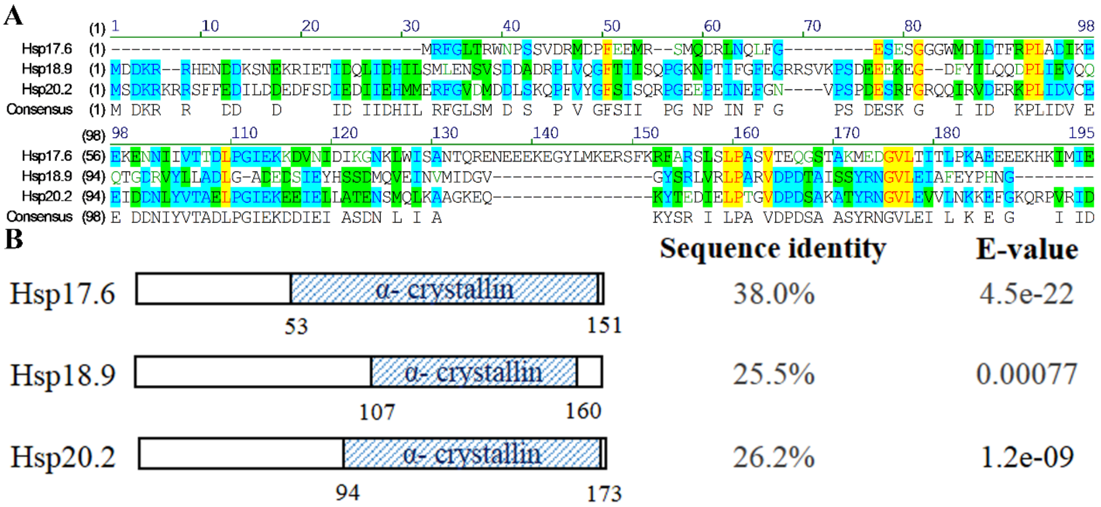

Figure S1. Protein homolog analysis of the three sHsps from *M. psychrophilus*. **(A).** Protein sequence alignment of Hsp17.6, Hsp18.9 and Hsp20.2 showed the identical (red letters) and consensus residues. Sequence alignment was preformed using the software Vector NTI. (**B**). A schematic showing the α-crystallin domains and locations within Hsp17.6, Hsp18.9 and Hsp20.2. The sequence identity and E-value of Hsp17.6 were compared with Pfam: HSP20, and those of Hsp20.2 and Hsp18.9 were compared with Pfam: ArsA_HSP20 because the two exhibit too low identities with HSP20.

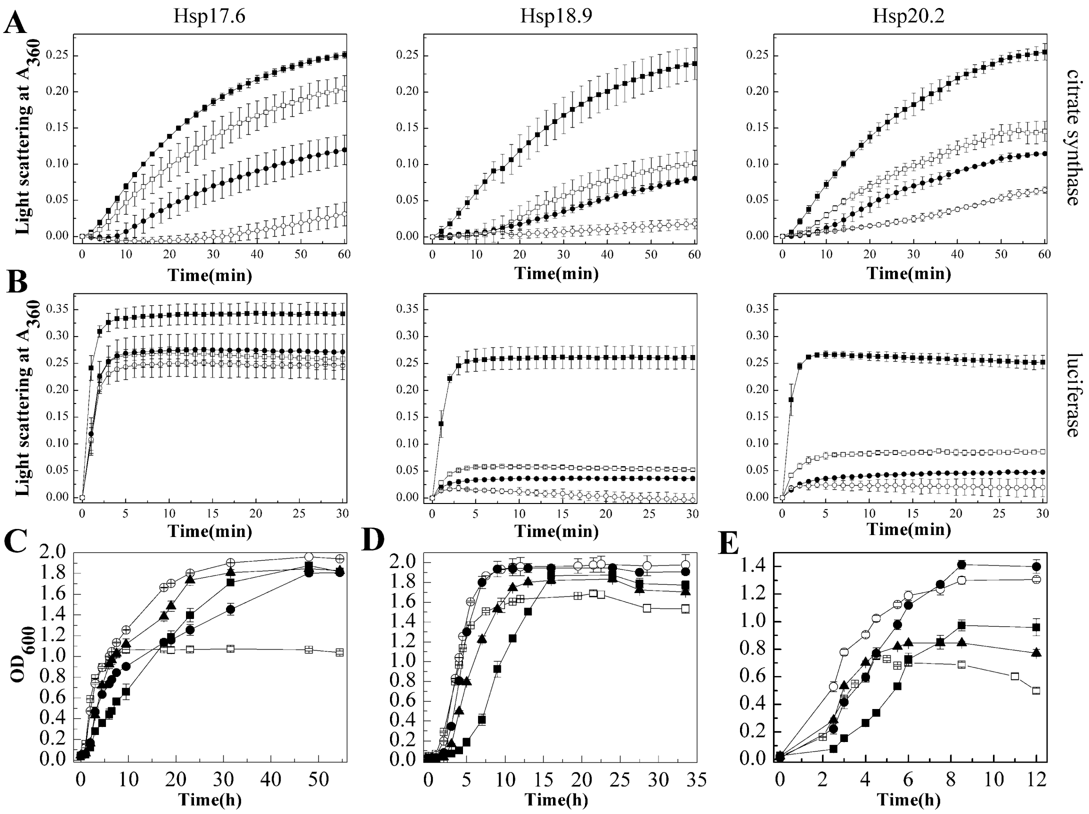

Figure S2. Assays of the three archaeal Hsps in suppression of heat-induced protein aggregations and effect on stationary cell density of the heterologously expressed *E. coli*. Citrate synthase (CS, **A**) and Luciferase (**B**) were used as the tested substrates. The CS monomer (600 nM, **A**) and luciferase (1 μM, **B**) were each incubated with 0 (■), 1.2 μM (●), 2.4 μM (▲), or 12 μM (▼) of Hsp17.6 (left), Hsp18.9 (middle) or Hsp20.2 (right), respectively. Protein mixtures were incubated at 45 ℃, and light scattering at 360 nm were continuously monitored within 60 min. (**C-E**). Hsp17.6 (○), Hsp18.9 (●), Hsp20.2 (▲) and the *E. coli* IbpA/B (■), or empty vector pET28a (□) were each expressed in *E. coli* JM109 (DE3), and the strains were cultured at 22 ℃ (**C**), 37 ℃ (**D**) and 45 ℃ (**E**), respectively. Growths were monitored by measurement of OD_{600}. Three independent experiments were performed, and the averages and standard deviations are shown.

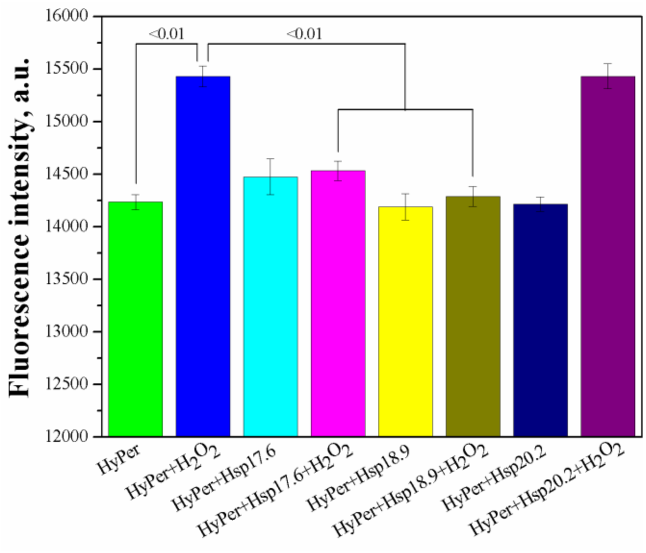

Figure S3. Fluorescence intensity changes of HyPer in 1 mM H_2O_2 with or without addition of 4:1 molar ratio of the archaeal sHsps. Protein of 25 µg/mL in TE buffer was assayed for the fluorescence using BioTek's Synergy H4 Hybrid Reader. Excitation was provided at 500 nm, and emission was collected from a range of 545 nm. Three independent experiments were performed, and the averages and standard deviations are shown. Difference significances of H_2O_2 treated Hyper fluorescence intensity in the presence and absence of sHsps were statistical analyzed by the ANOVA and Tukey multiple comparison test.

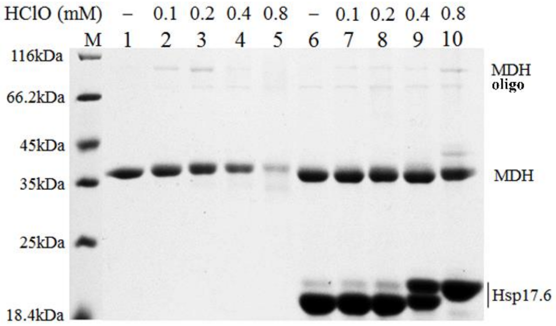

Figure S4. Effect of Hsp17.6 on MDH oxidation by different concentrations of HClO. MDH was each oxidized with 0 − 0.8 mM HClO at 37 ℃ for one hour, and then added with 4:1 molar ratio of Hsp17.6 (lane 6 − 10), and analyzed on non-reducing SDS-PAGE after 30 min treatment. Protein markers and the tested proteins are shown at the left and right of the gels, respectively.

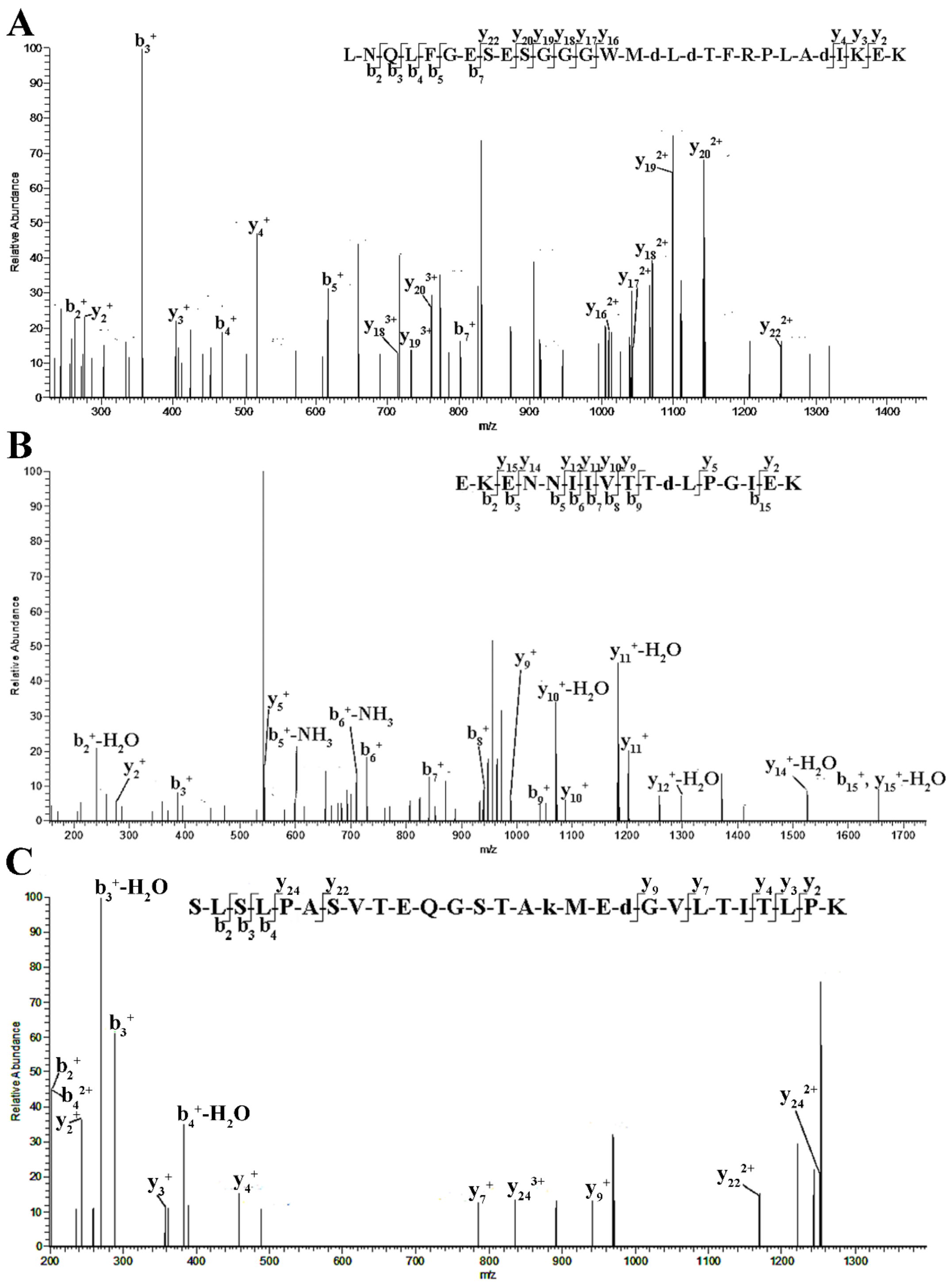

Figure S5. The representative MS/MS spectra showing the mass increased residues in oxidants treated Hsp17.6. The large protein bands on non-reducing SDS-PAGE were sliced and subjected to LC-MS/MS analysis as described in materials and methods. Insert in each MS/MS spectrogram showed the graphical fragment map of the relevant peptide sequence matching the observed fragmentation ion. Amino acids of lowercase

letters indicate oxidation of a molecular weight increment of 15.99492 Da. (**A**). Peptide fingerprint of D44-Oxidation (15.99492 Da), D46-Oxidation (15.99492 Da) and D53-Oxidation (15.99492 Da). (**B**). Peptide fingerprint of D66-Oxidation (15.99492 Da). (**C**). Peptide fingerprint of K129-Oxidation (15.99492 Da) and D132-Oxidation (15.99492 Da).

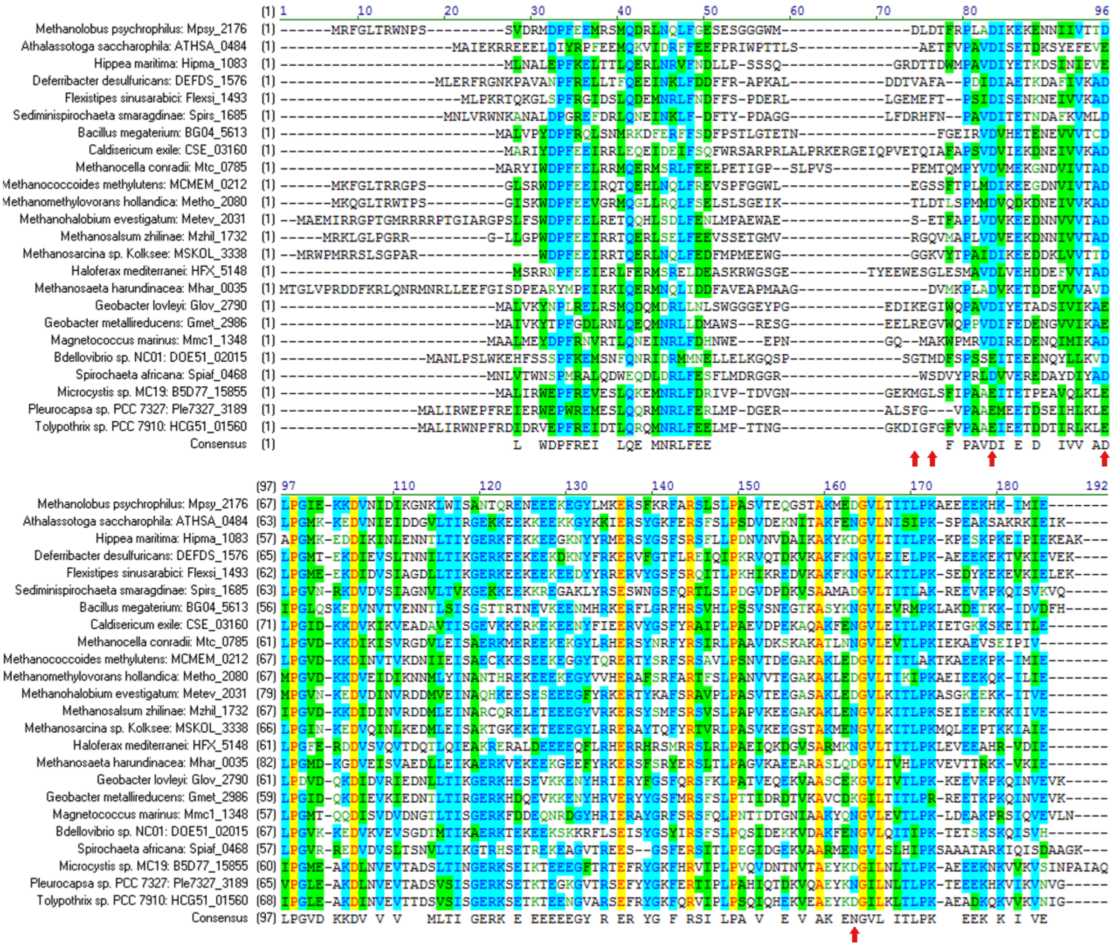

Figure S6. Protein sequence alignment of the small heat shock proteins of the *M. psychrophilus* Hsp17.6 orthologs. Hsp17.6 orthologs were retrieved from KEGG database and the protein accession numbers and the organisms from which the proteins were retrieved are shown. Sequence alignment was preformed using the software Vector NTI. Arrows indicate the residues that were mutated.

Table S1. Mass spectrometry detected residues percentages (%) of HClO- and H_2O_2-oxidized of larger and smaller Hsp17.6 bands, and HClO-oxidized MDH in the presence or absence of Hsp17.6*.

Residues	-	HClO (0.4 mM)	H_2O_2 (1 mM)
Hsp17.6	smaller	larger	larger
N9	8.04±1.10	26.35±3.15#	37.67±7.47#
M16	32.07±6.44	86.92±8.35#	74.51±3.08#
M22	66.06±15.73	86.78±7.48	53.71±5.01
M25	47.25±6.51	35.23±10.23	100.00±0.00#
D44	13.80±2.40	39.28±7.05#	46.74±13.17#
D46	15.92±3.90	23.95±4.02	24.30±6.56
D53	5.80±0.47	15.15±0.26#	9.68±2.48
N60	4.84±0.56	12.30±2.19#	5.08±3.82
D66	11.82±3.33	21.30±12.49	19.44±2.15
K72	0.46±0.27	5.02±0.31#	3.46±1.89#
M103	48.10±3.30	69.60±5.96	69.08±11.60
K104	33.05±4.91	45.29±6.57	29.33±6.75
M130	82.49±3.58	88.51±8.68	82.17±3.20
D132	1.53±0.02	6.40±2.80#	7.67±1.184#
MDH	-	HClO (0.4 mM)	HClO(0.4 mM) + Hsp17.6
K6	10.12±2.71	6.54±0.29	6.55±1.14
K7	12.69±2.42	16.30±1.47	14.12±3.03
F15	5.76±1.46	6.84±1.38	5.15±2.06
F22	4.24±2.47	3.71±2.15	1.47±0.53
K27	6.37±1.64	9.57±0.36#	5.69±2.43#
D31	14.15±0.19	18.49±3.50	13.19±0.28
N42	2.22±1.18	1.34±0.00	1.59±0.82
K45	4.72±0.34	11.23±4.96#	15.20±0.97
M51	1.49±0.13	13.10±0.26#	4.040±0.2#
N63	1.04±0.59	21.82±0.54#	4.042±0.5#
M91	2.04±0.00	72.84±0.74#	42.16±0.74#
D94	5.47±0.41	2.50±1.64	29.70±9.00

D95	50.76±1.14	50.95±0.08	33.33±0.00#
N100	2.89±1.62	9.66±4.14	8.64±0.55
K102	9.02±3.01	9.06±2.16	9.00±1.34
M104	63.83±18.00	80.18±0.77#	74.92±9.70
K105	29.09±10.91	26.38±2.11	18.21±4.88
N117	6.27±1.75	14.47±3.41#	6.69±2.70#
M130	15.24±1.12	73.28±14.58#	14.92±4.97#
F135	1.98±0.48	1.11±0.22	7.44±3.21
D153	5.24±0.75	6.61±0.09	4.85±1.21
H180	6.47±0.87	14.19±7.48#	7.30±.319#
D182	44.52±21.52	39.46±25.90	1.02±0.23
M184	11.77±3.09	91.74±6.64#	22.91±2.93#
P186	1.52±0.20	7.88±0.87#	12.64±7.02
D208	8.68±3.76	6.18±3.22	2.31±0.00
Y231	26.79±5.35	81.25±18.75#	42.43±24.25#
Y265	7.60±0.09	4.35±0.29	3.72±0.29
Y269	39.30±0.38	43.22±0.43	26.79±8.31
K294	41.14±2.39	41.43±0.74	42.02±1.79
K299	1.05±0.26	1.64±0.21	1.80±0.40
K305	46.83±8.73	56.31±1.76#	55.09±1.76
M308	33.60±46.37	68.59±14.74#	41.39±1.38#

* Protein bands were sliced from the non-reduced SDS-PAGE gel in Figure 3, and subjected to MS/MS analysis. Through comparing the relative abundances of the oxidized peptide fragments in H_2O_2- and HClO-pulsed vs. the non-pulsed Hsp17.6 and MDH, the over-representative oxidized residues were identified. Triplicate protein samples were subjected to MS/MS analysis, and the averages and standard deviations are shown. #, data are significantly different was verified by the ANOVA and Tukey multiple comparison test were comparation of non-treated proteins and oxidants-treated proteins of Hsp17.6 and MDH, and HClO-treated MDH in the presence and absence of Hsp17.6 ($P < 0.05$).

Table S2. Primers used in this study

Primer	Sequence (5’-3’)*	purposes
SMT3-F	ACCACCAATCTGTTCTCTGTG	Forward primer in amplification of pET28a-SMT3.
SMT3-R	CTCGAGCACCACCACCACCA	Reverse primer in amplification of pET28a-SMT3.
SUMO-0075-F	ACAGAGAACAGATTGGTGGT *ATGGACGATAAAAGACGTCATG*	Forward primer in amplification of Hsp18.9.
SUMO-0075-R	GTGGTGGTGGTGCTCGAG*TCA CCCGTTATGAGGATACTC*	Reverse primer in amplification of Hsp18.9.
SUMO-0869-F	ACAGAGAACAGATTGGTGGT*A TGTCAGATAAAAGAAAGAGGCG A*	Forward primer in amplification of Hsp20.2 gene.
SUMO-0869-R	GTGGTGGTGGTGCTCGAG*TCA ATCTATCCGGACTGGCCT*	Reverse primer in amplification of Hsp20.2 gene.
SUMO-2176-F	ACAGAGAACAGATTGGTGG T*ATGAGATTTGGACTTACAAG AT*	Forward primer in amplification of Hsp17.6 gene.
SUMO-2176-R	GTGGTGGTGGTGCTCGAG*T TATTCTATCATTATCTTGTGTT T*	Reverse primer in amplification of Hsp17.6 gene.
pET28a-ULP1-F	CTGAACGCTAGCCTTGTTC CTGAATTAAATGAAAAAG	Forward primer in amplification of ULP1 gene.
pET28a-ULP1-R	CGATAGCTCGAGCTATTTT AAAGCGTCGGTTAA	Reverse primer in amplification of ULP1 gene.
28a-F	CATGGTATATCTCCTTCTTA	Forward primer in amplification of pET28a.
28a-R	CTCGAGCACCACCACCACCA	Reverse primer in amplification of pET28a.
pET28a-2176-F	TAAGAAGGAGATATACCATG*A GATTTGGACTTACAAGATGGAA*	Forward primer in amplification of Hsp17.6 gene.
pET28a-2176-R	TGGTGGTGGTGGTGCTCGAG*T TCTATCATTATCTTGTGTTTT*	Reverse primer in amplification of Hsp17.6 gene.
2176-D46N-F	GGAGGATGGATGGATCTT**AAC** ACATTCCGACCTCTTGCC	Forward primer in amplification of D46N mutant of Hsp17.6 gene.
2176-D46N-R	GGCAAGAGGTCGGAATGT**GT**	Reverse primer in amplification of D46N

	**T**AAGATCCATCCATCCTCC	mutant of Hsp17.6 gene.
2176-D53N-F	CATTCCGACCTCTTGCC**AAC**A TCAAAGAGAAGGAGAAC	Forward primer in amplification of D53N mutant of Hsp17.6 gene.
2176-D53N-R	GTTCTCCTTCTCTTTGAT**GTT**G GCAAGAGGTCGGAATG	Reverse primer in amplification of D53N mutant of Hsp17.6 gene.
2176-D66N-F	CAATATAATCGTCACCACA**AA** **C**CTTCCCGGCATCGAGAAGAA	Forward primer in amplification of D66N mutant of Hsp17.6 gene.
2176-D66N-R	TTCTTCTCGATGCCGGGAAG**G** **TT**TGTGGTGACGATTATATTG	Reverse primer in amplification of D66N mutant of Hsp17.6 gene.
2176-D132N-F	TCAACTGCCAAAATGGA**AAC** GGTGTGCTCACGATCACAC	Forward primer in amplification of D132N mutant of Hsp17.6 gene.
2176-D132N-R	GTGTGATCGTGAGCACACC**GT** **T**TTCCATTTTGGCAGTTGA	Reverse primer in amplification of D132N mutant of Hsp17.6 gene.
2176-D44&46N-F	AGTGGTGGAGGATGGATG**AAT** CTT**AAC**ACATTCCGACCTCTT GCC	Forward primer in amplification of D44/46N mutant of Hsp17.6 gene.
2176-D44&46N-R	GGCAAGAGGTCGGAATGT**GT** **T**AAG**ATT**CATCCATCCTCCAC CACT	Reverse primer in amplification of D44/46N mutant of Hsp17.6 gene.

*, Italicized bases indicate homologous sequences gene fragment. Bold bases indicate the amino acid site of a point mutation.
